# Modulation of immune checkpoint regulators in interferon γ induced urothelial carcinoma and activated T-lymphocyte cells by cytostatics

**DOI:** 10.1038/s41435-023-00203-0

**Published:** 2023-05-03

**Authors:** Jörg Hänze, Johannes Schulte-Herbrüggen, Rainer Hofmann, Axel Hegele

**Affiliations:** 1 Department of Urology, Philipps-University Marburg, Marburg, Germany; 2 Department of Radiotherapy and Radiooncology, Faculty of Medicine, Philipps-University Marburg, Marburg, Germany; 3 Urological Center Mittelhessen, DRK Hospital Biedenkopf, Biedenkopf, Germany

**Keywords:** Translational immunology, Gene regulation in immune cells

## Abstract

Exploring the regulation of co-inhibitory (PD-1, PD-L1, CTLA-4) and co-stimulatory (CD28) genes by chemotherapeutic drugs is important for combined immune checkpoint blockade (ICB) therapy. ICB interferes with T-cell receptor and major histocompatibility complex (MHC) signaling by antibody drugs directed against the co-inhibitors. Here, we analyzed urothelial (T24) cell line with respect to cytokine signaling by interferon γ (IFNG) and the leukemia lymphocyte (Jurkat) cell line with respect to T-cell activation as mimicked by phorbolester and calcium ionophore (pma/iono). Alongside, we considered possible intervention with the chemotherapeutics gemcitabine, cisplatin and vinflunine. Noteworthy, cisplatin significantly induced PD-L1-mRNA in naïve and IFNG treated cells whereas gemcitabine and vinflunine had no effect on PD-L1-mRNA. At the protein level, PD-L1 showed typical induction in IFNG treated cells. In Jurkat cells, cisplatin significantly induced PD-1-mRNA and PD-L1-mRNA. Pma/iono administration did not alter PD-1-mRNA and PD-L1-mRNA but significantly increased CTLA-4-mRNA and CD28-mRNA levels where vinflunine suppressed the CD28-mRNA induction. In sum, we demonstrated that certain cytostatic drugs being relevant for the therapy of urothelial cancer, affect co-inhibitory and co-stimulatory modulators of immune signaling with potential impact for perspective combined ICB therapy of patients.

**Figure:**
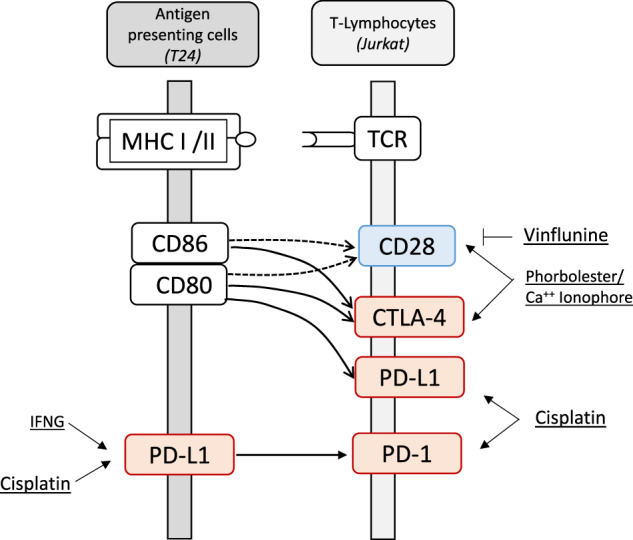
MHC-TCR signaling between antigen presenting cells and T-lymphocytes with co-stimulator (blue) and co-inhibitors (red) and interacting proteins (blank). Co-inhibitory connections are shown by lines and co-stimulatory connections by dotted lines. The inducible or suppressive actions of the drugs (underlined) on the respective targets are indicated.

MHC-TCR signaling between antigen presenting cells and T-lymphocytes with co-stimulator (blue) and co-inhibitors (red) and interacting proteins (blank). Co-inhibitory connections are shown by lines and co-stimulatory connections by dotted lines. The inducible or suppressive actions of the drugs (underlined) on the respective targets are indicated.

## Introduction

The immune checkpoint blockade therapy (ICB) targets signaling between T-cell receptor (TCR) and major histocompatibility complex (MHC) by antibody drugs and is applied for the treatment of a growing number of malignancies including urothelial carcinoma [1]. The immune response of T-cells is balanced through crosstalk of co-inhibitors such as PD-1, PD-L1, CTLA-4 and co-stimulator CD28 that signals between antigen-presenting cells or neoantigen-harboring tumor cells and T-cells involving CD80 and CD86 as further interacting molecules. Co-inhibitors counteracts co-stimulators and shift T-lymphocytes from the activation state towards the anergy and exhaustion state [2]. In those states, the immune responses become downregulated in the tumor microenvironment or local lymph nodes [3]. ICB drugs reverse the co-inhibition and restore T-cell effector function. PD-1, CTLA-4 and CD28 are expressed predominantly in T-lymphocytes. PD-L1 is found in higher abundance in tumor cells and antigen presenting cells [2]. Obviously, the regulation of the corresponding genes for PD-1, PD-L1, CTLA-4 and CD28 by cytostatic drugs is of potential interest when combining ICB and chemotherapy.

In this scenario, we focused on chemotherapeutic drugs that are recommended for the treatment of urothelial cancer such as cisplatin, vinflunine and gemcitabine [4]. As cell targets, we analyzed particularly the urothelial cell line (T24) and the immune cell leukemia T-lymphocyte (Jurkat) cell line. In T24 cells, cytokine signaling by interferon γ (IFNG) [5] and in Jurkat cells phorbolester/ionomycine (PMA/Iono) a mimic for T-cell activation were tested for interference with the cytostatic drugs [6].

## Materials and methods

Adherent urothelial cell lines and the suspension T-cell derived Jurkat cell line Jurkat, (DSMZ No. ACC282) were cultured according to protocols by DSMZ, Braunschweig, Germany and as decribed [7]. In subsets, IFNG (10 ng/ml) (R&D Systems) was added to urothelial T24 cells (DSMZ no.: ACC 376) and phorbol 12-myristate 13-acetate (pma) (Sigma) (100 nM) with ionomycin calcium salt (iono) (Sigma) (100 nM) to Jurkat cells [6] for 24 h. The chemotherapeutic drugs were added for 24 h in doses based on literature: Gemcitabine hydrochlorid (1 µM) [8], Cis-Diamminelplatinum (II) dichloride (100 µM) [9], Vinflunine (ChemScene) 10 (µM) [10]. Procedures of RNA and protein isolation, quantitative real time RT-PCR and Western-blot has been described previously by our laboratory [7]. The DNA sequences of forward (+) and reverse primers (−) are subsequently listed: PD-L1 (CD274) (+) GCGAATTACTGTGAAAGTCAATGCC, (−) TGGTCACATTGAAAAGCTTCTCCTC; PD1 (PDCD1) (+) GGCCGCACGAGGGACAATAG, (−) AGGAAAGACAATGGTGGCATACTCC; STAT1 (+) ATGATGAACTAGTGGAGTGGAAGCG, (−) CTCTGAATGAGCTGCTGGAAAAGAC; CD28 (+) ATTGGGCAATGAATCAGTGACATTC, (−) AAGCTATAGCAAGCCAGGACTCCAC; CTLA4 (+) AACCTCACTATCCAAGGACTGAGGG, (−) AGCATTTTGCTCAAAGAAACAGCTG; β-actin (ACTB) (+) TATCCAGGCTGTGCTATCCCTGTAC, (−) TTCATGAGGTAGTCAGTCAGGTCCC. The antibodies were as follows: PD-L1 #13684 (Cell signaling), PD-1, host goat (AF1086, R&D Systems); (LDHA #3558, Cell signaling). Data were analysed with MS-Office Excel and Graphpad Prism Version 9.50.

## Results

**Fig. 1:**
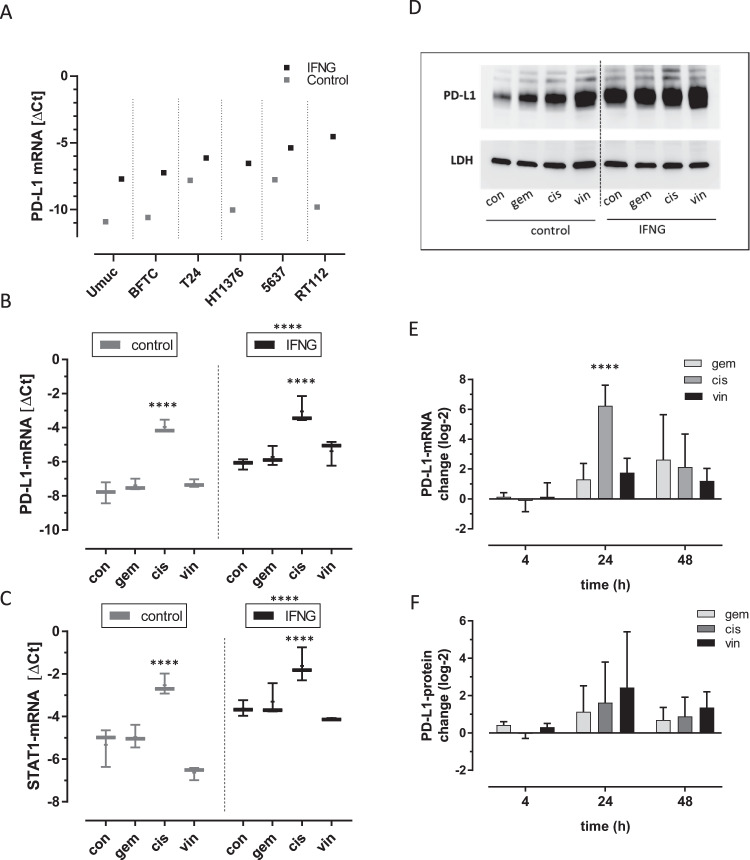
Regulation of PD-L1 signaling in urothelial carcinoma cells. **A** Screening of PD-L1-mRNA (ΔCt) levels in control (grey square) and interferon γ (IFNG; black square) treated cell lines (UMUC3, BFTC905, T24, HT1376, 5637, RT112). PD-L1-mRNA (**B**) and STAT1-mRNA (**C**) in T24 cells upon treatment with IFNG and cytostatic drugs (cemcitabine, gem; cisplatin, cis; vinflunine, vin) versus controls (con) that were combined in two subsets. **D** Western-blot analysis of PD-L1 (~50 kd) with loading control LDHA (~37 kd) from T24 samples treated as indicated. Time kinetic of changes of PD-L1-mRNA (**E**) and PD-L1 protein (**F**) upon treatment with cytostatic drugs in T24 cells. Significances were determined by 2-way-ANOVA with subsequent multiple comparison (****p < 0.0001). Data are displayed as box and whiskers with all values and mean.

Screening of several urothelial cell lines displayed different levels of PD-L1-mRNA each with typical induction by IFNG (Fig. 1A). For further analysis of the chemotherapeutic drugs, we selected the T24 cells with high basic levels of PD-L1-mRNA. Cisplatin significantly induced PD-L1-mRNA in naïve and IFNG treated cells whereas gemcitabine and vinflunine had no effect on PD-L1-mRNA in both groups (Fig. 1B). In accordance, the IFNG signaling mediator STAT1-mRNA matched the changes of PD-L1-mRNA both in the control and IFNG treated cells and by cisplatin (Fig. 1C). At the protein level, PD-L1 showed typical induction in the IFNG treated samples. Adding cisplatin or vinflunine lead to further PD-L1 accumulation in the control group and in the IFNG treated group (Fig. 1D). The time kinetic revealed strongest upregulation of PD-L1-mRNA by cisplatin and vinflunine after 24 h (Fig. 1E). Similarly, PD-L1 protein peaked at 24 h of cisplatin or vinflunine treatment but here the effects on PD-L1 protein appeared stronger with vinflunine (Fig. 1F).

**Fig. 2:**
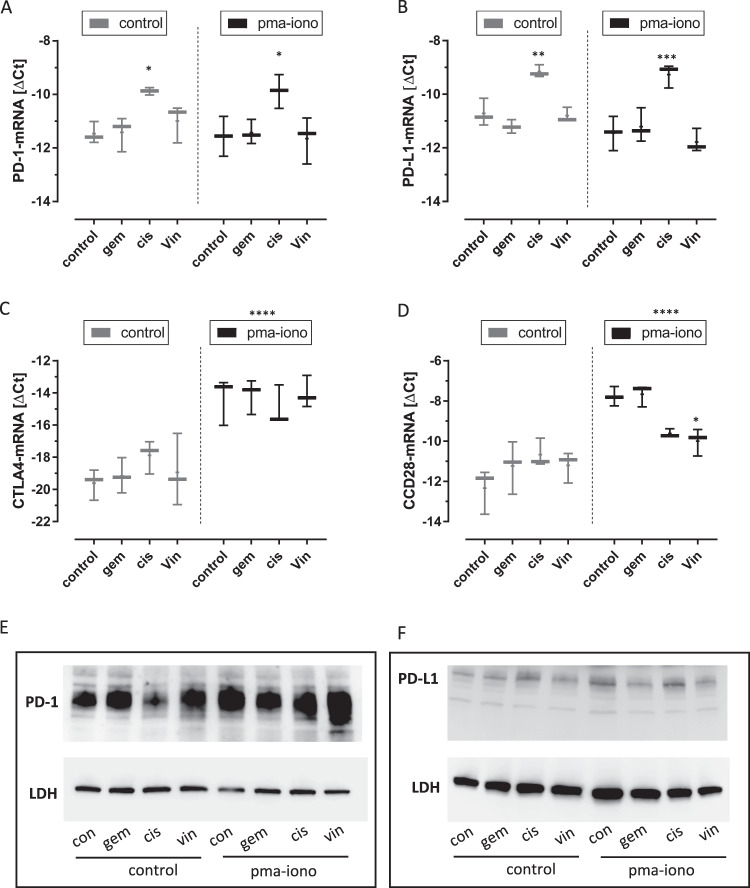
Regulation of immune checkpoint components in Jurkat cells. PD-1-mRNA (**A**), PD-L1-mRNA (**B**), CTLA4-mRNA (**C**) and CD28-mRNA (**D**) upon treatment with phorbolester/Ca ++ Ionophore (pma/iono) and cytostatic drugs (gemcitabine, gem; cisplatin, cis; vinflunine, vin) versus controls (con) that were combined in two subsets. Protein analysis by Western-blot of PD-1 (~40 kd) (**E**) and PD-L1 (~50 kd) (**F**) with loading control LDHA (~37 kd) treated as indicated. Significances were determined by 2-way-ANOVA with subsequent multiple comparison (*p < 0.05, **p < 0.01; ***p < 0.001; ****p < 0.0001). Data are displayed as box and whiskers with all values and mean.

Next, we analyzed Jurkat cells treated by gemcitabine, cisplatin and vinflunine. We tested control cells and pma/iono treated cells for mimicking T-cell activation. Cisplatin exerted significant induction of PD-1-mRNA and PD-L1-mRNA whereas Pma-iono treatment did not result in changes of PD-1-mRNA and PD-L1-mRNA (Fig. 2A, B). In addition, we analyzed the co-inhibitor CTLA-4 and the co-stimulator CD28 (Fig. 2C, D). Strikingly, pma-iono treatment significantly increased CTLA-4-mRNA and CD28-mRNA levels whereas, gemcitabine, cisplatin and vinflunine had no effect on CTLA4-mRNA. Of note, vinflunine suppressed the pma-iono induced increase of CD28-mRNA. In addition to mRNA data, an exemplary Western blot of PD-1 and PD-L1 (Fig. 2E, F) reveal that PD-1 protein appears stronger than PD-L1 protein particularly when comparing the Jurkat PD-L1 protein level (Fig. 2F) with those in T24 cells (Fig. 1D).

## Discussion

This study investigated combined effects of IFNG signaling and T-cell activation with cytostatic drugs for gene regulation of immune checkpoint modulators.

### Chemotherapeutic drugs and immune signaling in cancer and T-cells

The cisplatin effects on PD-L1 observed here add to related studies performed on various malignancies. Cisplatin induced PD-L1-mRNA in lung cancer cells and in tumor tissue of cisplatin treated patients [11]. As a relevant mechanism, our data suggest that cisplatin acts to some extent via STAT1 the crucial downstream mediator of IFNG signaling [5]. The subsequent consequences of pro-inflammatory IFNG signaling for tumor progression are multipart since diverse actions meet [12] and must therefore be seen in the specific context of disorder. IFNG enhances MHC-I thereby favoring neo-antigen presentation of tumor cells. Conversely, immune escape is facilitated by induction of immune checkpoints. Furthermore, IFNG dependent induction of cell cycle arrest, as well as, apoptosis have been reported.

Apart from IFNG signaling, several other pathways have been demonstrated to induce PD-L1. The cGAS/STING pathway has been assigned a critical role for cisplatin- induced PD-L1 in ovarian cancer [13]. A downstream arm of cGAS/STING pathway converge with the NF-kb pathway that targets PD-L1 promoter as well [14]. In a further study, cisplatin dependent PD-L1 induction has been attributed to the ERK1/2 and AP1 signaling pathway as demonstrated in several urothelial cell lines [15].

In the Jurkat T-cell model, the induction of CD28 and CTLA4 during T-cell activation [3] could be mimicked by activation of PKC pathway and intracellular Ca^++^ accumulation supporting this pharmacologic intervention as relevant trigger. The cisplatin-dependent upregulation of PD-1-mRNA along with PD-L1-mRNA in Jurkat cells, defines targets related to different signaling pathways. For PD-1 induction, IL-2 and TGF-β1 signaling [16] were demonstrated as relevant rather than IFNG for PD-L1 [5] were demonstrated as relevant. The selective downregulation of CD28-mRNA by vinflunine in Jurkat cells indicates a further branch possibly affecting combined or sequential therapy outcomes.

Of note, a recent experimental study compared combined therapy of anti-PD-1 therapy with either cisplatin or gemcitabine in lung and pancreatic cancer models and patients tissue samples [17]. Interestingly, cisplatin but not gemcitabine acted synergistically with PD-1 blockade therapy by increased T cell infiltration with release of antitumor cytokines involving the triggered cGAS/STING pathway. The cisplatin enhanced PD-1-mRNA levels, as observed here in the Jurkat T-cell model, may mechanistically add to the therapeutic beneficial effects in that study [17] since higher PD-1 levels may favor anti-PD-1 blockade therapy.

### Platinum-based drugs in urothelial cancer

As a platinum-based drug, we focused on cisplatin that is the most common applied drug member from the first generation. Alternatively, carboplatin is employed for advanced urothelial cancer patients who are ineligible for cisplatin. Carboplatin displays less systemic toxicity and is administered in patients with poor performance status such as those with restricted renal function. Pharmacologically, cisplatin and carboplatin bind to DNA via intra- and interstranded crosslinks causing DNA damage thereby triggering cell cycle block and apoptosis. Chemically, carboplatin has a ‘slower leaving group release’ when reacting with nucleophiles such as N7-guanine in DNA and this is attributed to less myelosuppressive related side effects. The common pharmacologic action of cisplatin and carboplatin suggests similar regulation of PD-L1 and PD-1. Variations in “non-canonical” actions targeting molecules beyond DNA, on the other hand, could differentially affect PD-L1 and PD-1 expression, an as yet undefined and speculative mechanism [18].

### Combined and sequential (maintenance) therapy by chemotherapy and ICB of patients with urothelial cancer

In a phase 3 trial of metastatic urothelial cancer (IMvigor130) [19], combined chemotherapy with ICB by atezolizumab displayed a favorable safety profile. Whereas, a benefit in terms of patients’ overall survival could not be demonstrated. In another trial of first-line therapy for advanced urothelial carcinoma (KEYNOTE-361), combined chemotherapy with pembrolizumab was not superior to chemotherapy alone in treatment efficacy [20]. Noteworthy, when applied sequentially after first line chemotherapy, ICB with avelumab significantly improved overall survival of patients with advanced urothelial and therefore ICB can be recommended as maintenance therapy (JAVELIN Bladder 100) [21]. To date, these studies have not definitively ruled out differences in treatment outcomes of patients with ICB between cisplatin and carboplatin.

The presented experimental data from cancer and immune cells may provide a hint as to how chemotherapeutic drugs can interfere with ICB. Most strikingly, cisplatin interfered with gene regulatory pathways that target immune checkpoints in cancer or immune cells and thereby is connected with ICB.

Restrictively, the time period considered in the cell culture studies (24 h) with combined addition of chemotherapeutics, interferon γ or pharmacologic T-cell activation is shorter than the time period (>weeks) of chemotherapeutic treatment of patients. Our cell experimental strategy aimed to simulate the tumor microenvironment. During tumor progression, intercellular signaling between tumor cells and infiltrating immune cells occurs dynamically and over an extended period of time. These apparent differences point to the limitations of this cell biology study.

In conclusion, we demonstrated that certain chemotherapeutic drugs that are relevant for the therapy of urothelial cancer can affect distinct co-inhibitory and co-stimulatory mediators of immune cell signaling [11]. Strikingly, among these are cisplatin and vinflunine. The observed shifts in mRNA levels of immune signaling proteins suggest possible impact on immune checkpoint blockage therapy that may be of importance for investigation of tumor models and relevant patients.

## Data Availability

All data generated or analysed during this study are included in this published article.
